# Effects of Physical Activity in the High School Curriculum on Cardiovascular Health, Cognitive and Physical Performance

**DOI:** 10.3390/jfmk8030101

**Published:** 2023-07-24

**Authors:** Tobias Jagomast, Theresa Mohr, Paul Niklas Axt, Kai Mortensen, Folke Brinkmann, Markus Weckmann, Gordon Ring, Michael Reppel, Daniel Drömann, Klaas F. Franzen

**Affiliations:** 1 Medical Clinic III, University Clinic Schleswig-Holstein Campus Luebeck, Ratzeburger Alle 160, 23562 Luebeck, Germany; tobias.jagomast@uksh.de (T.J.); themohr@t-online.de (T.M.); paul.axt@student.uni-luebeck.de (P.N.A.); daniel.dromann@uksh.de (D.D.); 2 Airway Research Center North (ARCN), German Center for Lung Research (DZL), 22927 Großhansdorf, Germany; folke.brinkmann@uksh.de (F.B.); markus.weckmann@uni-luebeck.de (M.W.); 3 Cardiology Kiel, 24116 Kiel, Germany; kaimortensen@yahoo.de; 4 Clinic for Rhythmology, Campus Lübeck, University Hospital Schleswig-Holstein, 23562 Lübeck, Germany; reppel@kardiologie-landsberg.de; 5 Section for Pulmonary Pediatrics, Campus Lübeck, University Hospital Schleswig-Holstein, 23562 Lübeck, Germany; 6 DRK Krankenhaus Mölln-Ratzeburg, Anästhesie, Röpersberg 2, 23909 Ratzeburg, Germany; g.ring@drk-krankenhaus.de; 7 Cardiology Landsberg, 86899 Landsberg, Germany

**Keywords:** arterial stiffness, pupils, students, prevention, sports, cognitive performance

## Abstract

Cardiovascular health at a young age has implications for preventing cardiovascular disease, and it is associated with improved physical and cognitive performance during the aging process. Sports are well known to prevent cardiovascular disease; however, school-based interventions have mostly been neglected. This cross-sectional study aimed to compare groups of high school students, stratified by the amount of physical activity in their high school curriculum and downtime. Comparisons concerning physical and cognitive performance and arterial stiffness were made. A total of 63 senior-year students were investigated. Arterial stiffness was assessed using the oscillometric technique with Arteriograph^TM^ detection. Three-kilometer and pendulum runs were conducted as typical training loads. Cognitive performance was evaluated via the visual and verbal memory and number connection tests. Regarding cognitive skills, extracurricular physical activity improved the number connection test in male participants (*p* = 0.004). For physical performance, female students with a sports-focused curriculum were faster in the 3 km run (*p* < 0.001). Concerning arterial stiffness, the measurements yielded a lower mean arterial pressure (*p* = 0.015) and aortic pulse wave velocity (*p* = 0.04) in male students with a sports-focused curriculum. In summary, extracurricular physical activity and enrollment in a sports-focused curriculum may be associated with lower cardiovascular risk due to lower arterial stiffness and better physical and cognitive abilities.

## 1. Introduction

In recent decades, the prevention, diagnosis, and treatment of cardiovascular diseases (CVDs) have become increasingly meaningful in patient care. CVDs are the most common chronic diseases of the 21st century, and their risk factors are highly prevalent in youths [1,2]. CVDs in adolescents have multidirectional implications.

First, CVDs and associated conditions still have high mortality [3,4]. Especially in industrial countries, overweight and obesity tend to occur early in childhood [5,6]. Other features of the CVD cluster are also widely prevalent in youths, such as pre-hypertension, pre-diabetes, and early development of atherosclerosis [7,8,9]. These medical conditions are assumed to be the first steps in the development of metabolic syndrome in adulthood [10].

Second, CVDs are associated with cognitive performance. In older subjects, it has been shown that even subclinical manifestations of CVDs are associated with impaired cognitive performance [11,12]. In young people, cardiovascular health is related to improved cognitive performance [13,14]. Looking into specific cognitive functions, a large study conducted by Crichton et al. revealed an association between visual-spatial memory, working memory, scanning and tracking, executive function, and cardiovascular health [15]. More generally a study by King et al. found that there is an overproportioned decline in fluid relative to crystallized intelligence in the presence of cardiovascular risk factors [16]. Reduced brain perfusion and chronic low-grade inflammation due to CVD risk factors are discussed as possible pathophysiological links [17,18].

Third, healthcare systems and providers are increasingly confronted with major logistical, financial, and medical challenges in CVD treatment [1,19]. Associated conditions and risk factors, among which are diabetes mellitus and obesity, require complex therapy and consume many resources [20].

Consequently, the prevention of CVDs has gained momentum. Advancements in this field are driven by politics, researchers, healthcare providers, and other players in the field. Harmful behaviors such as smoking and high-caloric diets act as independent risk factors and are widely prevalent in Western society [21,22,23]. Research has identified physical activity as one of the most important modifiable lifestyle factors to prevent CVDs and to also improve cognitive performance [24,25,26,27,28]. Early intervention has been linked to reduced CVDs in later adulthood [29,30]. Prevention programs in adolescents, therefore, comprise education, lifestyle interventions, and physical activity promotion [31,32].

One of the interventions at the intersection of education and physical activity is the restructuring of secondary education in German high schools. Since 2008/2009, students in Schleswig-Holstein can choose a focus in their curriculum, starting in grade 11 through to grade 13. This focus goes along with a set of adjacent subjects from a holistic approach. One of these focuses is sports. The overall benefits of sports, in general, have been extensively studied [33]. However, the objectifiable effects of participation in the so-called sports curriculum regarding cardiovascular health and physical and cognitive performance have not yet been investigated in Germany, and there are a lack of studies in the international context that confirm the hoped-for benefits of an increased frequency of school sports [34].

This study aimed to close the above-mentioned knowledge gap. In an exploratory cross-sectional study, different parameters were considered, including cardiovascular health, such as arterial stiffness, physical performance, and cognitive performance. Under the assumption of beneficial outcomes in participants in the sports curriculum, we investigated the aforementioned parameters and argue that physical activity in school is of great importance. The results might generate questions for future studies regarding the importance of school sports in the overall well-being of adolescents.

## 2. Materials and Methods

### 2.1. Data Collection

This study was a cooperation between three secondary schools (Luebeck, Bad Oldesloe, and Ratzeburg; Schleswig-Holstein, Germany), the Medical Clinic III of the University Hospital Schleswig-Holstein Luebeck, and the Ministry of Education and Science Schleswig-Holstein. Data were prospectively collected from April to May 2012. All schools within a reasonable area that offered sports curricula were asked to participate. A total of 63 senior-year students were finally enrolled, based on voluntary participation. An examination was carried out for hemodynamic parameters and physical and cognitive performance. Groups were stratified by their curriculum, which focused on sports, language, or science. The resulting groups were further defined as “sports curriculum students” (SCS) or “other curricula students” (OCS). SCS had five hours of physical education per week. In comparison, OCS attended only two hours of physical education per week. Furthermore, the cohort was dichotomized by extracurricular physical activity. More than 300 min of sports per week at a moderate intensity was termed as being physically active (PAS); otherwise, participants were classified as physically inactive (PIS). Based on the World Health Organization Guidelines (WHO), 150–300 min are recommended; however, a large majority of subjects (90.5%) fulfilled this criterion, which caused the adaption of the chosen cut-off for statistical analysis [25].

Data collection took place in total for one to two days at each school and was conducted by an attending doctor in internal medicine and one medical student. On day 1, all participants at each school first had to answer a questionnaire on their medical background, lifestyle habits, and type, duration and frequency of physical activity apart from high school. Following the first part of the study, we employed two standardized tests on the subjects’ cognitive skills. The time to conduct the number connection test (NCT) inversely correlates with the intelligence quotient (IQ) and displays perceptual speed [35,36]. The visual and verbal memory test (VVM) indicates associative memory [37]. In the second part, on day 1 or 2, depending on the number of participants at each school, physical performance was assessed using two tests. The pendulum run reflects speed, in which the distance between two targets 10 m apart must be covered as many times as possible within a 1 min interval. A 3 km run was carried out to test endurance. There was break of at least 120 min between the two tests. Before the tests, the subjects’ arterial stiffness at rest in a lying position was measured for at least 10 min using an Arteriograph^TM^ (TensioMed^TM^, Budapest, Hungary). Data for aortic pulse wave velocity (PWVao), systolic blood pressure (SBP), diastolic blood pressure (DBP), and mean arterial pressure (MAP) were collected [38,39,40,41].

### 2.2. Statistical Analysis

The statistical analysis was carried out using the pseudonymized data set with R [42]. All comparisons on continuous variables were performed using a Wilcoxon rank-sum test, since the data were imbalanced and non-normally distributed according to the Shapiro–Wilk test. Two-level ordinal variables were compared by Fisher’s exact test. For an overview of the coherences of the different variables, a correlation matrix was created and graphically depicted using Spearman’s rank correlation coefficient. Smoking and alcohol consumption were excluded from the inferential statistics due to the small percentages of smokers (7.9%) and heavy alcohol consumers (1.7%), which were defined as smoking or consuming alcohol multiple times per week.

We performed post-hoc power analysis on the given sample sizes. For the comparisons regarding cognitive testing, we assumed medium effect size, hence power for comparison of the entire cohort was 73%, respectively 41% for the female subgroup, and 38% male subgroup stratified by curriculum. When stratified by physical activity power was 37% for male and 45% for female subgroup. For comparisons of physical performance and hemodynamics, we assumed a large effect size yielding power of 82% for the female subgroup and 74% for the male subgroup.

## 3. Results

### 3.1. Characteristics of the Test Groups

**Figure 1:**
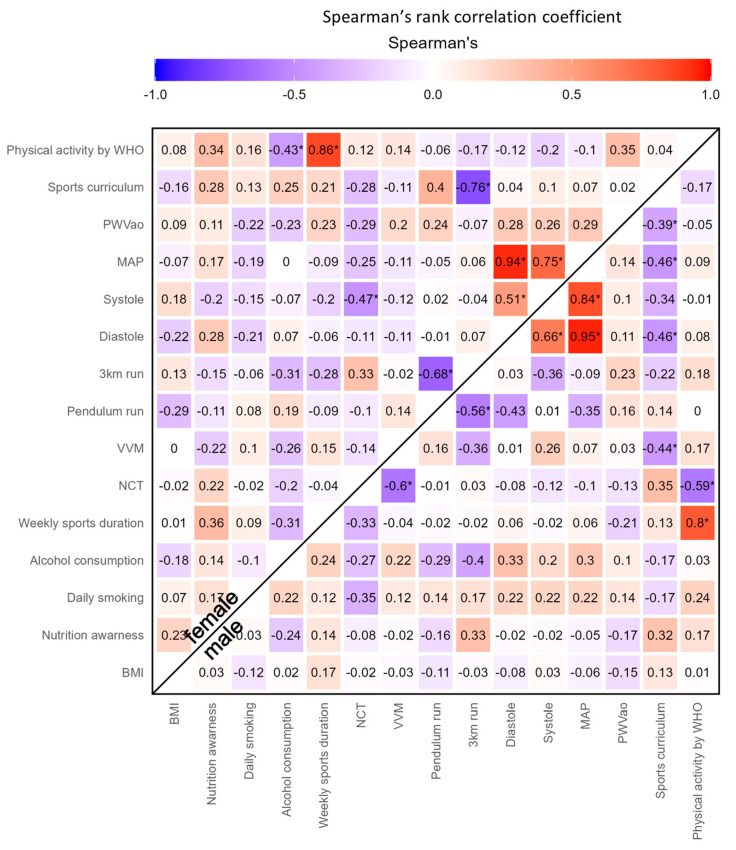
Correlation matrix of the investigated variables stratified by sex for overview (using Spearman’s rank correlation coefficient). Nutrition awareness and daily smoking were encoded as 1 for “yes” and 0 for “no”. Alcohol consumption had five levels: 5 = daily, 4 = weekly, 3 = weekends, 4 = monthly, 1 = less than monthly. School curriculum was encoded as 1 for SCS and 0 for OCS, while physical activity was encoded using WHO guidelines as 1 for ≥300 min per week (PAS) and 0 for <300 min per week (PIS). * *p* < 0.05.

Our cohort comprised 63 students, of which 33 were female and 30 were male (Table 1). Of these 63 students, 42 were enrolled in the sports curriculum and 21 in other curricula. Regarding extracurricular physical activity, 35 of the 63 students stated that they participated in sports for at least five hours a week and were assigned to the physically active group; 20 students did not fulfill this criterion and were termed physically inactive, while eight students did not provide information about sports performed in their downtime. The mean age of our cohort was 18.9 years. Their mean body mass index (BMI) was normal (22.32 kg/m^2^). There was a slight but significant difference between males and females, with males showing a higher BMI (*p* = 0.011). Half of the participants self-reported that they were aware of healthy nutrition. This effect was significant for sports curriculum students (SCS) compared with other curricula students (OCS) (*p* = 0.031), and was pronounced for physically active students (PAS) compared to physically inactive students (PIS) (*p* = 0.086). A large majority of the participants neither smoked nor consumed alcohol regularly, defined as daily and multiple times a week, respectively (Table 1). Interestingly, alcohol consumption was inversely correlated with physical activity (as defined in the Section 2) in the female cohort (Figure 1).

### 3.2. Cognitive Performance

Comparing the results of the number connection test (NCT), there was no significant difference between OCS and SCS. However, OCS showed significantly better performance in the visual and verbal memory test (VVM) (*p* = 0.044) (Table 2a). This difference was observed to be sex-specific in the male subgroup (*p* = 0.018) (Table 2b). Our analysis based on physical activity in general revealed no difference between PAS and PIS in either of the tests. Interestingly, a sex-specific comparison of male PAS and PIS students revealed significantly better performance by PAS in the NCT (*p* = 0.004) (Table 2d). No differences were observed between all male and female participants in the VVM or NCT.

### 3.3. Physical Activity

On average, the participants engaged in physical activity for approximately 450 min a week, with no significant difference between sexes. Interestingly, there was also no difference in the weekly sports duration of the SCS and OCS. Physical ability in the 3 km and pendulum runs was greater in the male participants (*p* < 0.001). Therefore, sex-stratified comparisons were carried out regarding OCS and SCS, as well as PAS and PIS, to avoid bias due to different sex ratios in these groups. For the male group, no difference was observed in the tests, either comparing OCS and SCS, or PAS and PIS. However, in the female subjects, SCS showed superior performance in the 3 km run (*p* < 0.001) (Table 3), while a comparison of female PAS and PIS yielded no significant difference.

### 3.4. Hemodynamics and Arterial Stiffness

Concerning the hemodynamic data (diastolic and systolic blood pressure (DBP and SBP), mean arterial pressure (MAP), and aortic pulse wave velocity (PWVao)) in the entire cohort, there was no difference according to sex, OCS, or SCS, nor PAS and PIS. However, in the male subcohort, SCS tended to have more favorable measurements for DBP (*p* = 0.015), MAP (*p* = 0.015), and PWVao (*p* = 0.04) (Table 4a). These differences were not observed when comparing PAS and PIS males. Female students showed no significant differences in either comparison.

## 4. Discussion

Cardiovascular risk factors include diabetes mellitus and arterial hypertension, but also modifiable behaviors such as alcohol and tobacco consumption and physical inactivity [43]. Today’s medicine increasingly focuses on the earliest detection and reduction of these risk factors. In addition to improved treatment of diabetes mellitus and high blood pressure, primary prophylactic measures are offered, creating incentives to change lifestyle, nutrition, and physical activity level [44]. This study was carried out under the assumption that increased physical activity in adolescents goes along with benefits to physical and mental performance and arterial vascular stiffness. The results can be summarized as follows.

Sex-wise, our cohort was balanced. However, we investigated twice as many SCS as OCS students. The participants showed normative BMI and overall high health awareness, with only a small proportion of smokers and regular alcohol consumers. There were no self-reported major health concerns present in the cohort. Interestingly, SCS had more nutrition awareness than OCS, possibly because SCS are more interested in nutrition to enhance physical performance and therefore chose their specific curriculum. Moreover, they are exposed to the theoretical background of physical performance and nutrition science in their classes. Other studies that have investigated this topic argue that, generally, athletes might get advice from their coaches and teammates, aside from formal education [45,46]. Still, studies about athletes’ nutrition knowledge are rare and largely descriptive. The underlying causes remain elusive thus far. On the other hand, studies show health benefits from nutrition intervention programs in school children [47,48]. This underlines the importance of establishing nutritional education in early childhood and adolescence.

Our examination of physical capability revealed no difference in weekly sports duration between SCS and OCS students. Since male students had better results on the 3 km and pendulum runs, we carried out sex-specific data analysis, because SCS and OCS had different sex ratios. SCS females were faster on the 3 km run than OCS females, while the status of physical activity in general showed no effect. In the male subcohort, neither curriculum choice nor stated general physical activity level influenced the results. Overall, SCS students did not show superior results on the physical tests. However, it must be considered that the comparison of weekly time spent in at least moderate-intensity sports between the SCS and OCS showed no significant difference. Apparently, students that lack physical education in school make up for it in their leisure time. Despite this, we hypothesize that the observed effect in SCS females might be due to the specific training. In our questionnaire, we assessed what specific sports the students participated in. Despite SCS and OCS spending the same amount of time engaged in physical activity per week, not all sports might prepare as well for a 3 km run (e.g., sports that demand less endurance). SCS students will train over this distance more often as part of the curriculum, thereby potentially acquiring a specific advantage.

In the subcohort of males, regarding cognitive performance, OCS outperformed SCS in terms of the VVM. Moreover, male PAS showed better results in the NCT than inactive males. For the female cohort, such results were not observed. It is possible that students enrolled in other curricula are more frequently challenged to memorize facts, since humanities and natural sciences make up a larger part of their study plan. However, we showed that physical activity in general goes along with improved scores in the NCT, confirming our hypothesis. Numerous studies have shown the positive effects of physical activity on cognitive function [27,28,49,50,51]. The results presented here suggest that the NCT in particular might be sensitive to the level of athleticism. The NCT measures perceptual speed, one aspect that underlines all qualities of intelligence and therefore correlates with IQ [36]. Perceptual speed plays a major role in sports [52]. In an elderly cohort, Oswald et al. showed superior improvements in cognitive tests such as the NCT when subjects received physical and cognitive training combined [53]. The sex-specific effect was previously observed by Legault et al. in adolescent athletes when testing the perceptual–cognitive abilities of the participants [52]. A biological cause still needs to be identified. However, Legault et al. argued that the investigated female athletes were not on an equal level of training compared to their male counterparts. Additionally, the type of sport plays a role in improving specific cognitive functions [54]. The VVM in our study, as a test of associative memory, seemed to be less affected by physical activity. Perhaps memory and recall play a minor role in the sports played by the cohort. Further research is warranted to investigate the relationships between specific qualities of intelligence and different kinds of sports.

Evaluation of arterial stiffness yielded significant differences only in the male subcohort. Male SCS had lower DBP, MAP, and PWVao. Due to their curriculum, these students regularly engage in endurance sports. Research shows that this type of physical activity has a positive effect on the arteries. Physical activity in general may therefore be of secondary importance, while the kind of sport is more relevant [55]. There is also a significant negative association between physical fitness and the occurrence of arterial hypertension in adolescents [56]. The risk of hypertension decreases as fitness level improve [57,58]. Other studies have proven that reduced physical activity and high BMI lead to an increase in PWV and thus to increased arterial stiffness [59,60]. Both results suggest that students would benefit from additional physical education classes. The sex-specific effect observed in our study has previously been reported. A review article by DuPont et al. summarized the current state of knowledge [61]. A priori, women have lower baseline PWV [62]. After short bursts of exercise, the effects on PWV are only observed in men [63,64]. Moreover, PWV inversely correlates with cardiorespiratory endurance, but only in men [65]. Men are believed to have greater testosterone exertion during exercise than women [66]. Testosterone is known to lower PWV in the short and long terms [67,68,69]. In their review article, Moreau et al. argued that these effects are due to structural changes in the endothelium, as well as acute changes in nitric oxide signaling [70,71]. Perhaps due to the elevated PWV baseline in men, changes due to exercise are more pronounced, caused by higher testosterone release, compared to women. However, most results were obtained by observational studies. Testosterone signaling pathways of vascular function have only been extensively characterized in preclinical models [70]. Further identification of corresponding human signaling pathways is warranted to link observations to biological mechanisms.

Our study has some limitations. A power analysis revealed insufficient power, less than 80%, especially for the subgroup comparisons of cognitive performance. However applicable sample size was limitedsince all eligible students within a reasonable regional area were included. Therefore, our study has pilot trial character, and statements are preliminary and further research is needed. Prospective analysis of a larger cohort, augmented by differentiated measures of physical and cognitive ability, would therefore be desirable. Additionally, questionnaires were employed to assess nutrition awareness and weekly time spent engaged in physical activity. A well-known bias of this approach is social desirability, which might have led to overestimating the actual time spent engaged in physical activity [72] as, in our study, nearly all students reported that they participated in at least 150 min of intermediate-to-high levels of physical activity per week, which is the amount recommended by the WHO.

## 5. Conclusions

The data reported showed the benefits of the German sports curriculum in terms of cardiovascular health and physical performance. Physical activity in general was associated with improved cognitive performance. However, due to the small number of subjects, this study is limited. Overall, enrollment in the sports curriculum correlated with performance on the 3 km run, DBP, MAP, and PWVao in a sex-specific manner. This might be due to specific endurance training, which is not a mandatory part of general physical activity. However, for cognitive performance on the NCT, the exact kind and nature of the sport seems of secondary importance, and better results were achieved by physically active students. To be able to demonstrate the long-term effectiveness of physical activity in schools on cardiovascular health in adolescents, a multi-center longitudinal study is warranted and different interventions should be compared. Moreover, research needs to focus on understanding underlying sex-specific changes in physiology caused by exercise.

## Figures and Tables

**Table 1:** Cohort characteristics by (**a**) sex, (**b**) curriculum, and (**c**) physical activity.

**(a) Sex**	**Female (n = 33)**	**Male (n = 30)**	**Total (N = 63)**	***p*-Value**
Age (years)				0.136
Mean (SD)	18.697 (1.630)	19.167 (0.648)	18.921 (1.274)	
Size (m)				<0.001
Mean (SD)	1.698 (0.062)	1.834 (0.072)	1.763 (0.096)	
Weight (kg)				<0.001
Mean (SD)	63.556 (11.905)	76.231 (7.620)	69.592 (11.876)	
BMI (kg/m^2^)				0.011
Mean (SD)	22.006 (3.881)	22.658 (1.869)	22.316 (3.084)	
Healthy nutrition				0.612
N-Miss	2	0	2	
No	14 (45.2%)	16 (53.3%)	30 (49.2%)	
Yes	17 (54.8%)	14 (46.7%)	31 (50.8%)	
Smoking (multiple times a week)				0.183
No	32 (97.0%)	26 (86.7%)	58 (92.1%)	
Yes	1 (3.0%)	4 (13.3%)	5 (7.9%)	
Alcohol (multiple times a week)				0.483
N-Miss	2	1	3	
No	31 (100.0%)	28 (96.6%)	59 (98.3%)	
Yes	0 (0.0%)	1 (3.4%)	1 (1.7%)	
**(b) Sports curriculum (all)**	**OSC (n = 21)**	**SCS (n = 42)**	**Total (N = 63)**	***p*-Value**
Age (years)				0.898
Mean (SD)	19.048 (0.384)	18.857 (1.539)	18.921 (1.274)	
Size (m)				0.443
Mean (SD)	1.748 (0.095)	1.771 (0.096)	1.763 (0.096)	
Weight (kg)				0.838
Mean (SD)	69.750 (13.381)	69.513 (11.221)	69.592 (11.876)	
BMI (kg/m^2^)				0.737
Mean (SD)	22.834 (4.223)	22.057 (2.341)	22.316 (3.084)	
Healthy nutrition				0.031
N-Miss	1	1	2	
No	14 (70.0%)	16 (39.0%)	30 (49.2%)	
Yes	6 (30.0%)	25 (61.0%)	31 (50.8%)	
Smoking (multiple times a week)				1.000
No	19 (90.5%)	39 (92.9%)	58 (92.1%)	
Yes	2 (9.5%)	3 (7.1%)	5 (7.9%)	
Alcohol (multiple times a week)				0.333
N-Miss	1	2	3	
No	19 (95.0%)	40 (100.0%)	59 (98.3%)	
Yes	1 (5.0%)	0 (0.0%)	1 (1.7%)	
**(c) Physical Activity (all)**	**PIS (n = 20)**	**PAS (n = 35)**	**Total (N = 55)**	***p*-Value**
Age (years)				0.160
Mean (SD)	19.200 (0.523)	18.771 (1.646)	18.927 (1.359)	
Size (m)				0.227
Mean (SD)	1.736 (0.098)	1.778 (0.096)	1.763 (0.098)	
Weight (kg)				0.274
Mean (SD)	66.412 (11.418)	71.263 (12.334)	69.499 (12.133)	
BMI (kg/m^2^)				0.612
Mean (SD)	21.923 (2.522)	22.530 (3.585)	22.309 (3.227)	
Healthy nutrition				0.086
N-Miss	2	0	2	
No	12 (66.7%)	14 (40.0%)	26 (49.1%)	
Yes	6 (33.3%)	21 (60.0%)	27 (50.9%)	
Smoking (multiple times a week)				0.285
No	20 (100.0%)	31 (88.6%)	51 (92.7%)	
Yes	0 (0.0%)	4 (11.4%)	4 (7.3%)	
Alcohol (multiple times a week)				1.000
N-Miss	0	2	2	
No	20 (100.0%)	32 (96.7%)	52 (98.1%)	
Yes	0 (0.0%)	1 (3.3%)	1 (1.9%)	

**Table 2:** Cognitive performance of the (**a**) entire cohort by curriculum, (**b**) male students by curriculum, (**c**) female students by curriculum, (**d**) male students by physical activity, and (**e**) female students by physical activity.

**(a) Curriculum (all)**	**OSC (n = 21)**	**SCS (n = 42)**	**Total (N = 63)**	***p*-Value**
NCT (s)				0.923
N-Miss	0	1	1	
Mean (SD)	58.469 (17.240)	55.005 (7.439)	56.178 (11.682)	
VVM (points)				0.044
Mean (SD)	35.643 (7.013)	30.833 (7.888)	32.437 (7.889)	
**(b) Curriculum (male)**	**OSC (n = 9)**	**SCS (n = 21)**	**Total (N = 30)**	***p*-Value**
NCT (s)				0.066
N-Miss	0	1	1	
Mean (SD)	52.829 (14.677)	57.273 (6.746)	55.894 (9.839)	
VVM (points)				0.018
Mean (SD)	37.333 (6.083)	30.524 (6.969)	32.567 (7.333)	
**(c) Curriculum (female)**	**OSC (n = 12)**	**SCS (n = 21)**	**Total (N = 33)**	***p*-Value**
NCT (s)				0.108
Mean (SD)	62.698 (18.390)	52.846 (7.577)	56.429 (13.240)	
VVM (points)				0.524
Mean (SD)	34.375 (7.643)	31.143 (8.876)	32.318 (8.474)	
**(d) Physical activity (male)**	**PIS (n = 8)**	**PAS (n = 18)**	**Total (N = 26)**	***p*-Value**
NCT (s)				0.004
N-Miss	1	0	1	
Mean (SD)	66.210 (10.686)	53.055 (7.690)	56.738 (10.334)	
VVM (points)				0.403
Mean (SD)	29.812 (6.871)	32.750 (7.735)	31.846 (7.471)	
**(e) Physical activity (female)**	**PIS (n = 17)**	**PAS (n = 12)**	**Total (N = 29)**	***p*-Value**
NCT (s)				0.535
Mean (SD)	57.454 (14.680)	53.620 (10.793)	55.867 (13.138)	
VVM (points)				0.465
Mean (SD)	33.265 (8.722)	31.125 (9.303)	32.379 (8.867)	

**Table 3:** Comparison of physical tests in (**a**) female students and (**b**) male students.

**(a) Curriculum (female)**	**OSC (n = 12)**	**SCS (n = 21)**	**Total (N = 33)**	***p*-Value**
Pendulum run (repetitions)				0.061
N-Miss	2	8	10	
Mean (SD)	18.600 (0.966)	19.462 (1.664)	19.087 (1.443)	
3 km run (s)				<0.001
N-Miss	1	4	5	
Mean (SD)	1203.848 (177.048)	970.398 (68.789)	1062.111 (167.016)	
**(b) Curriculum (male)**	**OSC (n = 9)**	**SCS (n = 21)**	**Total (N = 30)**	***p*-Value**
Pendulum run (repetitions)				0.626
N-Miss	7	9	16	
Mean (SD)	20.500 (2.121)	21.250 (0.754)	21.143 (0.949)	
3 km run (s)				0.286
N-Miss	3	3	6	
Mean (SD)	798.483 (67.569)	747.831 (88.150)	760.494 (85.075)	

**Table 4:** Comparison of hemodynamic measurements in (**a**) male students and (**b**) female students.

**(a) Curriculum (Male)**	**OSC (n = 9)**	**SCS (n = 21)**	**Total (N = 30)**	***p*-Value**
DBP (mmHg)				0.015
N-Miss	0	1	1	
Mean (SD)	74.222 (9.418)	64.450 (9.237)	67.483 (10.218)	
SBP (mmHg)				0.073
N-Miss	0	1	1	
Mean (SD)	135.333 (16.000)	122.350 (14.214)	126.379 (15.735)	
MAP (mmHg)				0.015
N-Miss	0	1	1	
Mean (SD)	94.778 (10.814)	83.750 (9.408)	87.172 (10.974)	
PWVao (m/s)				0.040
N-Miss	0	1	1	
Mean (SD)	7.600 (1.482)	6.410 (1.007)	6.779 (1.276)	
**(b) Curriculum (female)**	**OSC (n = 12)**	**SCS (n = 21)**	**Total (N = 33)**	***p*-Value**
DBP (mmHg)				0.815
N-Miss	0	1	1	
Mean (SD)	68.333 (8.845)	68.700 (7.035)	68.562 (7.624)	
SBP (mmHg)				0.585
N-Miss	0	1	1	
Mean (SD)	123.083 (14.761)	125.250 (7.663)	124.438 (10.698)	
MAP (mmHg)				0.682
N-Miss	0	1	1	
Mean (SD)	86.500 (10.140)	87.550 (5.916)	87.156 (7.629)	
PWVao (m/s)				0.907
N-Miss	0	1	1	
Mean (SD)	6.650 (1.286)	6.530 (1.008)	6.575 (1.102)	

## Data Availability

Due to privacy restrictions, the authors have omitted public data availability.

## Abbreviations

BMI	Body mass index
CVD	Cardiovascular diseases
DBP	Diastolic blood pressure
IQ	Intelligence quotient
MAP	Mean arterial pressure
NCT	Number connection test
OCS	Other curricula students
PAS	Physically active students
PIS	Physically inactive students
PWVao	Aortic pulse wave velocity
SBP	Systolic blood pressure
SCS	Sports curriculum students
VVM	Visual and verbal memory test
WHO	World Health Organization
